# The Role of *graRS* in Regulating Virulence and Antimicrobial Resistance in Methicillin-Resistant *Staphylococcus aureus*

**DOI:** 10.3389/fmicb.2021.727104

**Published:** 2021-08-16

**Authors:** Le Chen, Zihui Wang, Tao Xu, Hongfei Ge, Fangyue Zhou, Xiaoyi Zhu, Xianhui Li, Di Qu, Chunquan Zheng, Yang Wu, Keqing Zhao

**Affiliations:** ^1^Department of Otolaryngology-Head and Neck Surgery, Eye Ear Nose and Throat Hospital, Fudan University, Shanghai, China; ^2^Key Laboratory of Medical Molecular Virology (MOE/NHC/CAMS), Department of Medical Microbiology and Parasitology, School of Basic Medical Sciences, Shanghai Medical College of Fudan University, Shanghai, China; ^3^Department of Infectious Diseases, Huashan Hospital, Fudan University, Shanghai, China; ^4^National Clinical Research Center for Aging and Medicine, Huashan Hospital, Fudan University, Shanghai, China; ^5^State Key Laboratory of Genetic Engineering, School of Life Science, Fudan University, Shanghai, China; ^6^Key Laboratory of Medical Molecular Virology (MOE/MOH/CAMS) and Institutes of Biomedical Sciences, Shanghai Medical College, Fudan University, Shanghai, China; ^7^Department of Gynecology, Obstetrics and Gynecology Hospital of Fudan University, Shanghai, China; ^8^Department of Otolaryngology, The Third Affiliated Hospital of Wenzhou Medical University, Ruian, Zhejiang, China

**Keywords:** *Staphylococcus aureus*, *graRS*, virulence, biofilm, two-component signal transduction system

## Abstract

Methicillin-resistant *Staphylococcus aureus* (MRSA) is a common cause of both community- and hospital-associated infections. The antibiotic resistance and virulence characteristics of MRSA are largely regulated by two-component signal transduction systems (TCS) including the *graRS* TCS. To make a relatively comprehensive insight into *graRS* TCS in MRSA, the bioinformatics analysis of dataset GSE26016 (a *S. aureus* HG001 WT strain vs. the Δ*graRS* mutant) from Gene Expression Omnibus (GEO) database was performed, and a total of 563 differentially expressed genes (DEGs) were identified. GO analysis revealed that the DEGs were mainly enriched in the “*de novo*” IMP biosynthetic process, lysine biosynthetic process *via* diaminopimelate, and pathogenesis; and they were mainly enriched in purine metabolism, lysine biosynthesis, and monobactam biosynthesis in KEGG analysis. WGCNA suggested that the turquoise module was related to the blue module, and the genes in these two modules were associated with *S. aureus* virulence and infection. To investigate the role of *graRS* in bacterial virulence, a *graRS* knockout mutant (Δ*graRS*) was constructed using MRSA USA500 2,395 strain as a parent strain. Compared to the wild-type strain, the USA500Δ*graRS* showed reduced staphyloxanthin production, retarded coagulation, weaker hemolysis on blood agar plates, and a decreased biofilm formation. These altered phenotypes were restored by the complementation of a plasmid-expressed *graRS*. Meanwhile, an expression of the virulence-associated genes (*coa*, *hla*, *hlb*, *agrA*, and *mgrA*) was downregulated in the Δ*graRS* mutant. Consistently, the A549 epithelial cells invasion of the Δ*graRS* mutant was 4-fold lower than that of the USA500 wild-type strain. Moreover, on the *Galleria mellonella* infection model, the survival rate at day 5 post infection in the USA500Δ*graRS* group (55%) was obviously higher than that in the USA500 group (20%), indicating *graRS* knockout leads to a decreased virulence *in vivo*. In addition, the deletion of the *graRS* in the MRSA USA500 strain resulted in its increased susceptibilities to ampicillin, oxacillin, vancomycin, and gentamicin. Our work suggests that the *graRS* TCS plays an important role in regulating *S. aureus* virulence *in vitro* and *in vivo* and modulate bacterial resistance to various antibiotics.

## Introduction

*Staphylococcus aureus* (*S. aureus*) is a major Gram-positive pathogen causing both community-acquired and hospital-acquired infections (Tong et al., 2015). The prevalence of methicillin-resistant *Staphylococcus aureus* (MRSA) has aroused more concerns over the past decades because of their antibiotic resistance and virulence (Otto, 2013). *Staphylococcus aureus* has 16 two-component systems (TCSs), which are significant for bacteria and commonly used to sense and respond to environmental changes (Capra and Laub, 2012; Burgui et al., 2018; Villanueva et al., 2018; Yan et al., 2019). The TCSs have been implicated in stress conditions, pathogenesis, and essential cellular pathways (Groisman, 2016). The *graRS* (Glycopeptide Resistance Associated) TCS has been shown to play an important role in response to CAMPs (cationic antimicrobial peptides) and promoting resistance to CAMPS by controlling the expression of *mprF* and *dlt* operons (Howden et al., 2008; Falord et al., 2011; Jousselin et al., 2013; Muzamal et al., 2014; Keinhorster et al., 2019). The *graRS* is also involved in regulating the susceptibility to vancomycin and daptomycin in MRSA (Doddangoudar et al., 2011; Cafiso et al., 2012; Mensa et al., 2014; Muller et al., 2018). However, the role of *graRS* in the regulation of bacterial virulence remains to be determined.

With the development of high throughput microarray and RNA-seq technologies, transcription profiles data provided by researchers worldwide are valuable resources for data mining, which can provide clues for investigations of gene functions. In this study, bioinformatics analysis including Gene Ontology (GO), Kyoto Encyclopedia of Genes and Genomes (KEGG), protein–protein interaction (PPI) networks, and weighted gene co-expression network analysis (WGCNA) were applied to identify the potential genes and pathways associated with *graRS* in the dataset GSE26016 (Falord et al., 2011). To further investigate the role of *graRS* in *S. aureus*, the *graRS* knockout mutant was constructed using the MRSA USA500 strain as the parent strain. The virulence-related phenotypes measurements *in vitro*, cell invasion assay, and *Galleria mellonella* model were carried out to investigate the role of *graRS* in regulating *S. aureus* virulence.

## Materials and Methods

### Microarray Materials

Dataset GSE26016 downloaded from Gene Expression Omnibus (GEO) database was performed to screen the hub genes related to *graRS*.^1^ GSE26016 is based on GPL11308 (BaSysBio *Staphylococcus aureus* T1 385K array) platform and includes three *S. aureus* HG001 wild type (WT) strains and three Δ*graRS* mutant strains, which cultured to mid-exponential phase in Tryptone soya broth (TSB) with 50 μg/ml colistin.

### Differentially Expressed Genes Identification

The raw microarray data of GSE26016 was processed and normalized in R software (version 3.5.2, USA). Then, the “limma” package was utilized to select differentially expressed genes (DEGs) between Δ*graRS* mutant strains and WT strains. |Log_2_ FC| (fold change) > 1 and adj. *p* < 0.05 were considered as statistically significant.

### GO and KEGG Enrichment Analyses of DEGs

To further understand the function of the DEGs in GSE26016, the Database for Annotation, Visualization, and Integrated Discovery (DAVID) online tool were used to perform the GO and KEGG pathway analyses (Zeng et al., 2019). GO analysis consists of three items, cellular component (CC), biological processes (BP), and molecular function (MF). R software was applied to visualize the results.

### Protein–Protein Interaction Network Construction and Hub Genes Identification

The STRING (Search Tool for the Retrieval of Interacting Genes) was used to analyze the interactions between the DEGs.^2^ The cut-off criterion of interaction with a combined score was set as >0.4. Cytoscape software (version 3.7.1, UAS) was applied to construct a PPI network and visualize the results. Moreover, the top four significant modules were identified by MCODE (molecular complex detection), a plug-in of Cytoscape software (Bandettini et al., 2012).

### Co-Expression Network Analysis

To identify the potential function and clusters of highly correlated genes in GSE26016, the co-expression network was constructed by the “WGCNA” package in R software. Topological Overlap Matrix (TOM) was applied to detect gene modules. Modules were regarded as branches and hierarchical clustering results, which were cut by the Dynamic Tree-Cut algorithm.

### Bacterial Strains and Culture Media

To make a relatively comprehensive insight into the effect of *graRS* in *S. aureus*, USA500 2395 strain was selected for further experiments. *Staphylococcus aureus* USA500 2395 strain was provided by Prof. Ying Zhang at Johns Hopkins University, and *S. aureus* ATCC 29213 was provided by Dr. Jinxin Zheng at Fudan University. TSB (Oxoid, United States) was used for bacterial cultivation. Mueller-Hinton Broth (MHB) and cation adjusted Mueller-Hinton Broth (CAMHB) were applied for antibiotic susceptibility tests.

### Construction of the Gene Knockout Mutant and Complementation Strains

To further explore the role of *graRS* in *S. aureus* USA500 2395 strain, the *graRS* knockout mutant and complementation strains were constructed according to the method we previously reported (Bai et al., 2019). Briefly, the *graRS* genes in methicillin-resistant *S. aureus* USA500 2395 strain were deleted by homologous recombination using the pKOR-1, which is a temperature-sensitive vector. The regions flanking *graRS* gene were amplified by PCR and then inserted into the pKOR-1. The recombinant plasmid pKOR-*graRS* was transformed to the *Escherichia coli* DC10B strain and then into the *S. aureus* USA500 strain by electroporation. The USA500Δ*graRS* was verified by PCR and RT-PCR. The vector pRB475 was used for *graRS* complementation. The DNA fragments of the *graRS* genes were amplified by PCR and then inserted into the vector. To form the complementary strain USA500Δ*graRS*:: pRB475-*graRS* (P*graRS*), the plasmid was transformed by electroporation into the USA500Δ*graRS*.

### RNA Extraction and Quantitative Real-Time-PCR

Total RNA was extracted from the *S. aureus* USA500 strain, the *graRS* knock-out mutant USA500Δ*graRS*, and the *graRS* complementation strain USA500Δ*graRS*:: pRB475-*graRS*. According to the results of bioinformatics analysis and the direction of our laboratory research, genes related to *graRS*, β-lactam resistance, and virulence were verified by quantitative real-time (qRT)-PCR. RNA extraction and qRT-PCR were carried out according to the previously established protocol (Bai et al., 2019). Briefly, overnight cultured USA500, USA500Δ*graRS*, and USA500Δ*graRS*:: pRB475-*graRS* were diluted 1:200 with TSB and incubated at 37°C with shaking (180 rpm) to an OD_600_ of 0.6 (4 h). Bacterial cells were collected and washed three times with ice-cold normal saline and homogenized using a Mini-BeadBeater-16 (Biospec, United States). The bacterial RNA was purified by using a RNeasy kit (Qiagen, Germany). DNase I-treated RNA was reversed to cDNA by using PrimeScript RT Master Mix (Takara, Japan). All samples were quantified by qRT-PCR with SYBR green reagents (Takara, Japan) and ABI 7500 real-time PCR system (Applied Biosystems, United States). The primers used in this study are shown in Table 1. The housekeeping gene *gyrB* was used as an internal control. All experiments were performed in triplicate and the relative expression level of mRNA was calculated by the 2^−ΔΔCT^ method.

**Table 1:** Primers used in this study.

Primers	Sequence (5' → 3')
gyrB(F)	ACATTACAGCAGCGTATTAG
gyrB(R)	CTCATAGTGATAGGAGTCTTCT
graR(F)	GTTGCTGGTATTGAAGATT
graR(R)	GTTCCATACTCATCACTTG
graS(F)	TTACTATATGAATGGTCTCGTAT
graS(R)	ACCTGACTAATATGTCTTGTT
vraF(F)	AAGAAGTGTTGCGAGATA
vraF(R)	ATGCTTGCGTATATCAGA
vraG(F)	GTTAATCGGTGTCGCTTAT
vraG(R)	ATCCTGTGGTAATCAATACTATAC
dltX(F)	GCCACCTAATAAATATGTTGAAGC
dltX(R)	GTGTCGCCACTGCCATAA
dltA(F)	GAAGGTGAACTTGTTATCG
dltA(R)	TTCCATTCTGTAGCCATT
mprF(F)	CGCTATTACTTCTGGCTTACG
mprF(R)	AATAATCTCCTCGCAATCTTCAAT
mecA(F)	TTAATAAGTGAGGTGCGTTA
mecA(R)	TAGGTGTTGGTGAAGATATAC
mecR1(F)	GATTAAGGCATTCCGACAA
mecR1(R)	CGACTACGACAGTTGGTA
femA(F)	GCTGAAGATGCTGGTGTAGTT
femA(R)	GTGCGGTATATGCTGCGTAA
femB(F)	TTCTCTGGTGGTTCATCA
femB(R)	CGCCATAATCTTCACTGTT
pbp1(F)	AAGTGCCAGATGTTGAAGG
pbp 1(R)	GTGTGCCAGAACCAATAGTAA
pbp 2(F)	ACACCTCAATACACTATG
pbp 2(R)	GGATACTACCACTTACTG
pbp 3(F)	TTCAGCCGTATCCAACATT
pbp 3(R)	GTGCCAAGAGGTCGTATT
pbp 4(F)	TATGAACAATAAGTGCTAATCC
pbp 4(R)	GACCTCCAACTGTAGAAG
fmtA(F)	TAACCAATCCATTATTACAT
fmtA(R)	ACAACTACATACTTATCATTA
fmtB(F)	ATTGCTAATGCTTCAGTT
fmtB(R)	ATAATGGTGTGGATAATGG
glmM(F)	TGGATTATGAGAGGCTGAA
glmM(R)	GAAGTGATGCGATTAGGTATT
fmtC(F)	CGCTATTACTTCTGGCTTACG
fmtC(R)	AATAATCTCCTCGCAATCTTCAAT
abcA(F)	GAACCTATTGAACCGACAGAA
abcA(R)	TTGGAACGACACATCATCTAAT
trfA(F)	GCATATCATAGCAATCCAAT
trfA(R)	ACTTCTGTTCTGTCTGTT
llm(F)	CTGCCTTAGTAGTTGCTT
llm(R)	CTATTGTAATGAGTCCGATTG
flp(F)	CCAATGATTCGCAACAACA
flp(R)	CAACATCTTGATAACCATAACCTT
prfp(F)	AACGCTTGTTGTAAGATT
prfp(R)	TGTTGTAAGTCAATTAGAGG
mdh(F)	GCACTTCCAATTACTGTT
mdh(R)	GCAGGTAGACTATATCGT
spx(F)	GTTGATATTGATTCACTACCA
spx(R)	CGTTGTGCTTCTTGTAAT
mgrA(F)	TACCTAATAAGCGATTAAGTT
mgrA(R)	AACGAATGGAACAAGTAG
norG(F)	GCACCAGCAATAAGAATAGG
norG(R)	TTCAACATCTCATATACAACCATT
sigB(F)	TTCCATTGCTTCTAACACTT
sigB(R)	GATGAACTAACCGCTGAAT
agrA(F)	GCAGTAATTCAGTGTATGTTCA
agrA(R)	TATGGCGATTGACGACAA

### Carotenoid Pigment Measurement

Colonies of *S. aureus* USA500, USA500Δ*graRS*, and USA500Δ*graRS*:: pRB475-*graRS* were cultured in TSB at 37°C for 12 h with shaking (180 rpm). The bacterial cells were collected by centrifugation (10,000 × *g*, 2 min), and washed three times with double distilled water. To extract carotenoids, 100% methanol was added to the cell pellets and then heating in a water bath at 55°C for 5 min. The supernatant containing extracted carotenoid pigment was quantified by measuring the OD_462_ values with a microplate reader (BioRAD, United States).

### Coagulase Test

The coagulase test was carried out using the freeze-dried rabbit plasma (Haibo, China). The overnight cultured *S. aureus* USA500, USA500Δ*graRS*, and USA500Δ*graRS*::pRB475-*graRS* were adjusted to the same OD_600_ value, and 0.8 ml suspension of each strain was added to the tubes, respectively. The tubes were shaken slightly to make sure the freeze-dried rabbit plasma dissolved completely. Afterward, they were incubated at 37°C and checked for visual clot formation every half hour until up to 6 h.

### Hemolytic Phenotype Analysis

The *S. aureus* USA500, USA500Δ*graRS*, and USA500Δ*graRS*::pRB475-*graRS* strains were inoculated on blood agar plates (BioMérieux, France) and cultured at 37°C for 48 h. Then the results of the hemolytic phenotype of these three strains were observed and photographed.

### Detection of Bacterial Biofilm Formation With the Microplate Assay

The overnight culture of bacterial strains was diluted 1:200 with TSB which supplemented with 1% glucose, and then added to a 96-well polyethylene microplate (200 μl bacterial suspension in each well). After incubation at 37°C for 24 h and 48 h, the biofilms were washed three times with phosphate-buffered saline (PBS) after removal of non-adhered bacteria. To fix the adhered cells, each well was added with 200 μl of 100% methanol. After the methanol was removed and the biofilms were stained with 2% crystal violet, the wells were washed with running tap water. The OD_570_ values of stained biofilms were measured by a microtiter-plate reader (Beckman Coulter, United States).

### Observation of *Staphylococcus aureus* Biofilms by Confocal Laser-Scanning Microscopy

The effect of *graRS* knockout on the *S. aureus* biofilms was evaluated by LIVE/DEAD staining. The bacterial strains were cultivated in TSB supplemented with 1% glucose in glass-bottomed fluorodishes for 48 h. The biofilms were washed with PBS 3 times, stained with 1 μM of SYTO9 and 1 μM of propidium iodide (PI) for 20 min, and then visualized by confocal laser-scanning microscopy (CLSM) with a 63× 1.4-NA oil immersion objective (Leica TCS SP8 Confocal Laser Scanning Platform, Leica Microsystems, Germany). The three-dimensional biofilm images were generated with IMARIS 7.0 software (Bitplane, United States). The thicknesses of the biofilms and the fluorescence intensities were determined using Leica Application Suite 1.0 software (Leica Microsystem).

### Antimicrobial Susceptibility Testing

The susceptibilities of *S. aureus* USA500, USA500Δ*graRS*, and USA500Δ*graRS*:: pRB475-*graRS* strains to various antibiotics were measured by the broth microdilution method based on the protocols of the American Clinical and Laboratory Standards Institute (CLSI). A 2-fold microdilution assay was performed to measure the minimal inhibitory concentrations (MICs) of the antibiotics. The range of the antibiotic’s concentrations was from 8 to 0.125 mg/L in the 96-well microplates containing CAMHB. Overnight cultured bacteria were diluted 1:200 with MHB and incubated at 37°C for 4 h. Then the 0.5 McFarland bacterial suspension was inoculated 1:200 into the CAMHB (for oxacillin, CAMHB+2% NaCl) and incubated at 35°C for 16 h or 24 h. *S. aureus* ATCC 29213 was included as controls.

### Epithelial Cell Invasion Assay

Human alveolar epithelial cells A549 were cultured in DMEM media (Hyclone, United States), which was supplemented with 10% FBS (fetal bovine serum; Gibco, United States). One day before infection, cells were seeded in 24 wells plates (Costar, United States) and cultured overnight at 37°C with 5% CO_2_. The cells infected at a MOI (multiplicity of infection) of 10:1 were centrifuged at 500 × *g* for 3 min and then incubated at 37°C with 5% CO_2_ for 2 h. To kill the extracellular bacteria, the medium containing 40 μg/ml lysostaphin and 200 μg/ml gentamicin was added to each well, which was previously washed three times with 500 μl PBS. The plates were incubated at 37°C for 30 min, and then washed twice with 500 μl PBS. The 0.1% Triton X-100 was applied to lyse the monolayer cells. After 20 min incubation, the lysates were diluted with PBS and plated on TSA plates to count the number of the intracellular bacteria.

### Virulence Assessment in *Galleria mellonella* Model

*Galleria mellonella* (*G. mellonella*) larvae were used as an invertebrate infection model to investigate the virulence of USA500, USA500Δ*graRS*, and USA500Δ*graRS*:: pRB475-*graRS* strains referring to previous reports (Insua et al., 2013; Mannala et al., 2018). Briefly, a total of 100 healthy *G. mellonella* larvae weighing between 250 and 300 mg were selected and randomly divided into five groups (blank control, PBS control, USA500, USA500Δ*graRS*, and USA500Δ*graRS*:: pRB475-*graRS*; 20 *G. mellonella* larvae/group). Each bacterial suspension (10^5^ CFU/ml) and the PBS control were injected into the four groups of larvae, respectively. The liquid was injected to the last left proleg of each larva with a 10 μl Hamilton syringe. The *G. mellonella* larvae were incubated at 37°C and monitored daily for survival for 5 days.

### Statistical Analysis

The analyses of GSE26016 were performed by R software (version 3.5.2, United States). GraphPad Prism version 7.0 software (La Jolla, United States) was used for statistical analysis. Kaplan–Meier analysis was performed for the survival plots of *in vivo* infection. A log-rank test was carried out for comparison of survival curves.

## Results

### DEGs Between *Staphylococcus aureus* HG001 Wild-Type Strains and Δ*graRS* Mutant Strains

**Figure 1:**
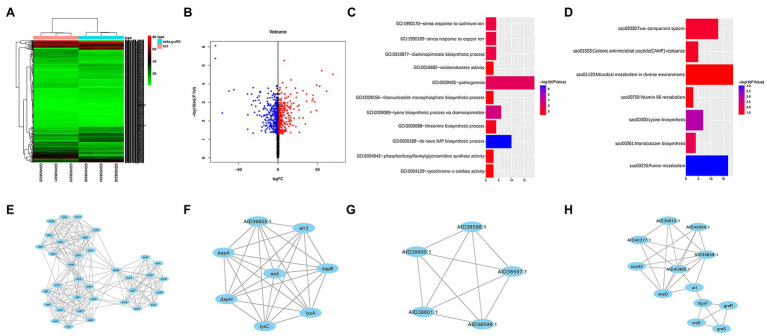
Bioinformatic analysis of GSE26016 dataset. **(A)** Heatmap representation of differentially expressed genes in *Staphylococcus aureus* HG001 wild-type strain and the Δ*graRS* mutant. The highly expressed genes of HG001 Δ*graRS* mutant strain compared with HG001 WT were represented by the red areas and the lowly expressed genes were represented by the green areas. **(B)** Volcano plot of the differentially expressed genes (DEGs) in GSE26016 identified by “limma” package in R software. Blue dots represent the down-regulated genes and red dots represent the up-regulated genes of HG001 Δ*graRS* mutant strain compared with HG001 WT. **(C)** Gene Ontology (GO) functional enrichment result of DEGs in GSE26016. **(D)** Kyoto Encyclopedia of Genes and Genomes (KEGG) functional enrichment result of DEGs in GSE26016. **(E–H)** Four significant modules in GSE26016 obtained by protein–protein interaction (PPI) of 563 DEGs: **(E)** The most significant module detected by MCODE (score = 13.176). **(F)** The second significant module detected by MCODE (score = 8). **(G)** The third significant module detected by MCODE (score = 5). **(H)** The fourth significant module detected by MCODE (score = 4.909).

GSE26016 includes three *S. aureus* HG001 WT strains and three Δ*graRS* mutant strains, which were grown with shaking (180 rpm) at 37°C in TSB containing 50 μg/ml colistin until OD_600_ of 1.0 was obtained. A total of 563 DEGs were identified by R software (adj. *p* < 0.05, |log_2_ FC| > 1), which were shown in the heatmap (Figure 1A). Among them, 269 genes were upregulated, and 294 genes were downregulated (Figure 1B).

### GO and KEGG Pathway Enrichment of DEGs Between *Staphylococcus aureus* HG001 Wild-Type Strains and Δ*graRS* Mutant Strains

GO and KEGG analyses were carried out by DAVID online tools to determine the biological functions of the 563 DEGs. GO analysis revealed that the DEGs were mainly enriched in “*de novo*” biosynthetic process of inosine monophosphate (IMP), lysine biosynthetic process *via* diaminopimelate, and pathogenesis; while in KEGG analysis they were mainly enriched in purine metabolism, lysine biosynthesis, and monobactam biosynthesis (Figures 1C,D).

### PPI Network of DEGs Between *Staphylococcus aureus* HG001 Wild-Type Strains and Δ*graRS* Mutant Strains

PPI network of DEGs was constructed based on the STRING database and the four most significant modules were obtained by MCODE. The IMP dehydrogenase *guaB* is relevant to metabolic and redox enzymes. The aspartate semialdehyde dehydrogenase (*asd*) is essential for the biosynthesis of lysine (Oogai et al., 2016). *lysC* is related to vancomycin resistance in *S. aureus* (Shoji et al., 2011). The *graR* and *graS* are relevant to *mprF* and *vraS*, and autolysin (*atl*) is involved in biofilm formation (Figures 1E–H; Herbert et al., 2007).

### Co-expression Network Analysis of GSE26016

There were 2,892 original mRNAs in GSE26016. After deletion and outlier values were eliminated, 2,825 original mRNAs remained. To group the coherent expression genes into modules, a hierarchical clustering algorithm was performed (Pu et al., 2020). Finally, 26 modules were obtained (Supplementary Figure S1A), and the number of mRNAs contained in the top six modules is shown in Table 2. Meanwhile, the blue module was related to the turquoise one (Supplementary Figures S1B,C). GO and KEGG analyses were performed in these two modules to further understand the correlation of these genes (Table 3).

**Table 2:** The number of mRNAs contained in different modules (top six modules).

Modules^*^	Freq^*^	Modules	Freq
Turquoise	964	Blue	497
Grey	407	Brown	332
Yellow	134	Green	52

* *Modules: Dynamic splicing of gene modules. Freq: The number of the gene modules*.

**Table 3:** GO and KEGG enrichment analyses of module blue and turquoise constructed by WGCNA (*p* < 0.05, count ≥ 10).

Module	Term	Count	*p*
Turquoise_GO	GO:0006189~“*de novo*” IMP biosynthetic process	11	0.002
	GO:0019843~rRNA binding	22	0.004
	GO:0022625~cytosolic large ribosomal subunit	16	0.005
	GO:0000287~magnesium ion binding	36	0.007
	GO:0003735~structural constituent of ribosome	29	0.012
	GO:0005737~cytoplasm	145	0.016
	GO:0051287~NAD binding	13	0.047
Turquoise_KEGG	sao01110: Biosynthesis of secondary metabolites	108	0.000
	sao00230: Purine metabolism	34	0.001
	sao01130: Biosynthesis of antibiotics	76	0.002
	sao00300: Lysine biosynthesis	11	0.004
	sao01100: Metabolic pathways	183	0.007
	sao01210:2-Oxocarboxylic acid metabolism	14	0.009
	sao01230: Biosynthesis of amino acids	51	0.010
	sao00290: Valine, leucine, and isoleucine biosynthesis	10	0.017
	sao00770: Pantothenate and CoA biosynthesis	11	0.031
	sao03018: RNA degradation	10	0.033
Blue_GO	GO:0005576~extracellular region	35	0.000
	GO:0009405~pathogenesis	28	0.000
	GO:0003735~structural constituent of ribosome	15	0.010
	GO:0009401~phosphoenolpyruvate-dependent sugar phosphotransferase system	11	0.010
	GO:0016020~membrane	11	0.034
Blue_KEGG	sao00052: Galactose metabolism	13	0.000
	sao05150: *Staphylococcus aureus* infection	11	0.010

### Effect of *graRS* Mutation in the MRSA USA500 Strain on Bacterial Virulence *in vitro*

**Figure 2:**
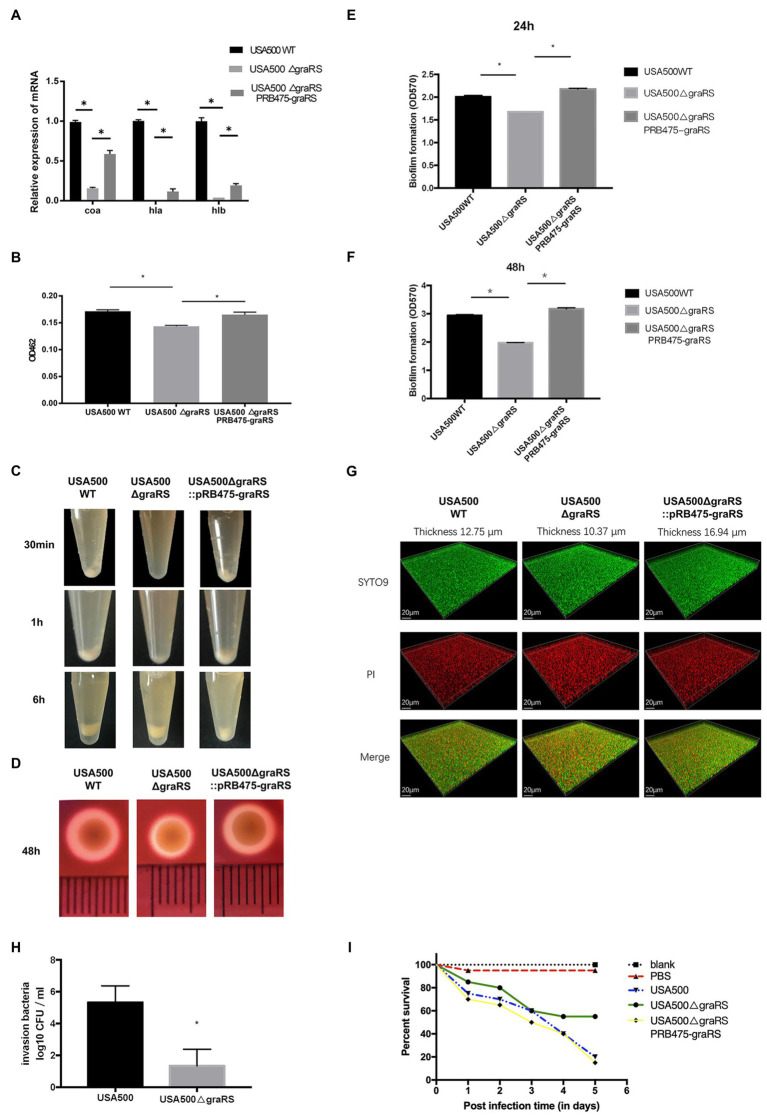
The effect of *graRS* knockout on the expression of the virulence-associated genes, virulence phenotypes and biofilm formation. **(A)** Transcriptional levels of the virulence genes in USA500WT, USA500Δ*graRS*, and USA500Δ*graRS::*pRB475-*graRS* strains were detected by qRT-PCR. **(B)** The effect of *graRS* deletion in the USA500 on the carotenoid pigment formation. **(C)** The effect of *graRS* knockout in the USA500 on the coagulase activities at 30 min, 1, and 6 h. **(D)** The effect of *graRS* FIGURE 2deletion in the USA500 on β-hemolysis on the blood agar at 48 h. **(E,F)** The microtiter plate assay was carried out to detect the ability to form biofilms of USA500WT, USA500Δ*graRS*, and USA500Δ*graRS::*pRB475-*graRS* strains in TSB supplemented with 1% glucose at 24 h **(E)** and 48 h **(F)**. **(G)** Confocal laser-scanning microscopy (CLSM) was performed to visualize the three-dimensional structure of biofilms formed by the three strains. The bacterial strains were cultivated in TSB supplemented with 1% glucose in glass-bottomed fluorodishes for 48 h. The biofilms were stained with 1 μM of SYTO9 and 1 μM of propidium iodide (PI) for 20 min, and then observed by CLSM with a 63× 1.4-NA oil immersion objective. The three-dimensional biofilm images were generated with IMARIS 7.0 software. **(H)** Epithelial cell invasion assay: Human alveolar epithelial cells A549 infected by USA500 or USA500Δ*graRS* at a MOI (multiplicity of infection) of 10:1 were centrifuged at 500 × *g* for 3 min and then incubated at 37°C with 5% CO_2_ for 2 h. Then the medium containing 40 μg/ml lysostaphin and 200 μg/ml gentamicin was used to kill the extracellular bacteria. The cells were lysed by 0.1% Triton X-100, and the intracellular bacteria were diluted with PBS and plated on TSA plates for counting. **(I)** Virulence assessment in the *Galleria mellonella* infection model: A total of 100 healthy *G. mellonella* larvae were randomly divided into five groups (20 *Galleria mellonella* larvae/group). Among them, three groups were injected with the bacterial suspension of USA500, USA500Δ*graRS*, or USA500Δ*graRS::*pRB475-*graRS* (10^5^ CFU/ml), respectively. Another group was injected with sterile PBS (PBS control). The last group did not receive any treatment (untreated control). The five groups of *G. mellonella* larvae were incubated at 37°C and monitored daily for survival for 5 days (*p < 0.05).

According to the bioinformatics analysis, the DEGs in GSE26016 were enriched in pathogenesis category in GO analysis (Figure 1C). Meanwhile, the blue module and the turquoise module in WGCNA were associated with *S. aureus* infection and virulence (Table 3). As we know, *S. aureus* produces numerous virulence factors, including coagulase, alpha and beta hemolysin, etc. (Lan et al., 2010). To investigate the effects of *graRS* on the expression levels of associated genes in the *graRS* knockout mutant of the USA500 2395 strain, qRT-PCR was applied. As shown Figure 2A, the expression of *coa*, *hla*, and *hlb* were significantly downregulated in the Δ*graRS* mutation strain (greater than 6-fold). In addition, the virulence-related phenotypes, such as the formation of staphyloxanthin, coagulase, and hemolysis were examined. Compared with USA500 wild-type strain, the USA500Δ*graRS* mutant showed a reduced staphyloxanthin production (*p* < 0.05), which was restored in the *graRS* complementation strain (Figure 2B). In the coagulase test, when rabbit plasma was incubated with each diluted bacterial suspension, respectively, for 0.5 h, there was obvious flocculation precipitation in the tubes with USA500 and the *graRS* complementation strains, while no precipitation was seen in the tube with USA500Δ*graRS*. Then the tube with USA500Δ*graRS* showed a little visible white precipitate at 1 h, which as much less than those in the tubes with USA500 and the *graRS* complementation strains. All three tubes had immobile clots formed at 6 h (Figure 2C). In the hemolysis test, a clear hemolytic ring with a diameter of larger than 7 mm was observed around the USA500 colonies on blood agar plates, whereas a smaller ring (a diameter of 5 mm) was formed around USA500Δ*graRS* colonies. The phenotype was restored in the *graRS* complementation strain (Figure 2D).

### Effect of *graRS* Mutation on Bacterial Biofilm Formation

To investigate the effects of *graRS* on *S. aureus* biofilm formation, a microtiter plate assay was performed to detect the biofilms of the USA500, USA500Δ*graRS*, and USA500Δ*graRS*::pRB475-*graRS* strains, using TSB supplemented with 1% glucose as the culture medium. After 24 h incubation, the biofilm of USA500Δ*graRS* (OD_570_ = 1.678 ± 0.00) was decreased compared to wild-type strain (OD_570_ = 2.009 ± 0.03), and *graRS* complementation restored the biofilm formation to the wild-type level (OD_570_ = 2.178 ± 0.02; Figure 2E). After 48 h incubation, biofilm produced by USA500Δ*graRS* (OD_570_ = 1.975 ± 0.01) was significantly less than its wild-type counterpart (OD_570_ = 2.944 ± 0.03) and was also restored by *graRS* complementation (OD_570_ = 3.171 ± 0.04; Figure 2F). Consistently, when biofilms of the USA500, USA500Δ*graRS*, and USA500Δ*graRS*::pRB475-*graRS* strains were cultured in TSB supplemented with 1% glucose in fluorodishes and were observed under CLSM with live/dead staining (Figure 2G), the thickness of the *graRS* mutant biofilm (10.37 μm) was less than that of the parent strain (12.75 μm), and the thickness was restored by complementation with pRB475-*graRS* (16.94 μm).

### Effect of *graRS* Mutation on the Bacterial Invasion of the Epithelial Cells

The ability of bacteria to invade epithelial cells partly reflects the virulence of these strains. To further identify the effect of *graRS* knockout on *S. aureus* virulence, the invasion capacity of the USA500 and USA500Δ*graRS* strains was evaluated by the gentamicin protection assay on A549 cells. Bacterial strains were separately inoculated into A549 cells at a MOI of 10, incubated for 2 h, and then gentamicin and lysostaphin were added to kill extracellular bacteria. It showed that the intracellular bacterial cells of the USA500 and the Δ*graRS* mutant were log_10_ CFUs of 5.333 ± 0.60 and 1.333 ± 0.60 (*n* = 3, *p* < 0.01), respectively, indicating that *graRS* knockout led to a 4-fold reduction in the cell invasion rate (Figure 2H).

### Effect of *graRS* Mutation on MRSA Infections in the *Galleria mellonella* Model

To further investigate the influence of *graRS* knockout on *in vivo* infection, the *G. mellonella* larvae infection model that has been widely applied in studies of microbial pathogenesis was employed (Jonsson et al., 2017). The *G. mellonella* larvae were divided into three infection groups (USA500, USA500Δ*graRS*, USA500Δ*graRS*:: pRB475-*graRS*) and two control groups (blank control and PBS control), with 20 *G. mellonella* larvae per group. *Galleria mellonella* larvae were incubated at 37°C and monitored daily for survival for 5 days. On day 1, 2, 3, 4, and 5, the survival rate in the USA500 infection group was 75, 70, 60, 40, and 20%, respectively, while that in the USA500Δ*graRS* infection group was 85, 80, 60, 55, and 55%, respectively. The reduction in survival of *G. mellonella* after 5 days in the USA500Δ*graRS* group was obviously lower than that in the USA500 group (*p* < 0.05). The survival rate in the *graRS* complementation group was similar to that in the wild-type group. The blank control (untreated group) and the PBS treated group (uninfected group) showed 100 and 95% survival rates at day 5, respectively (Figure 2I).

### Effect of *graRS* Mutation in USA500 on the Antimicrobial Susceptibilities

**Figure 3:**
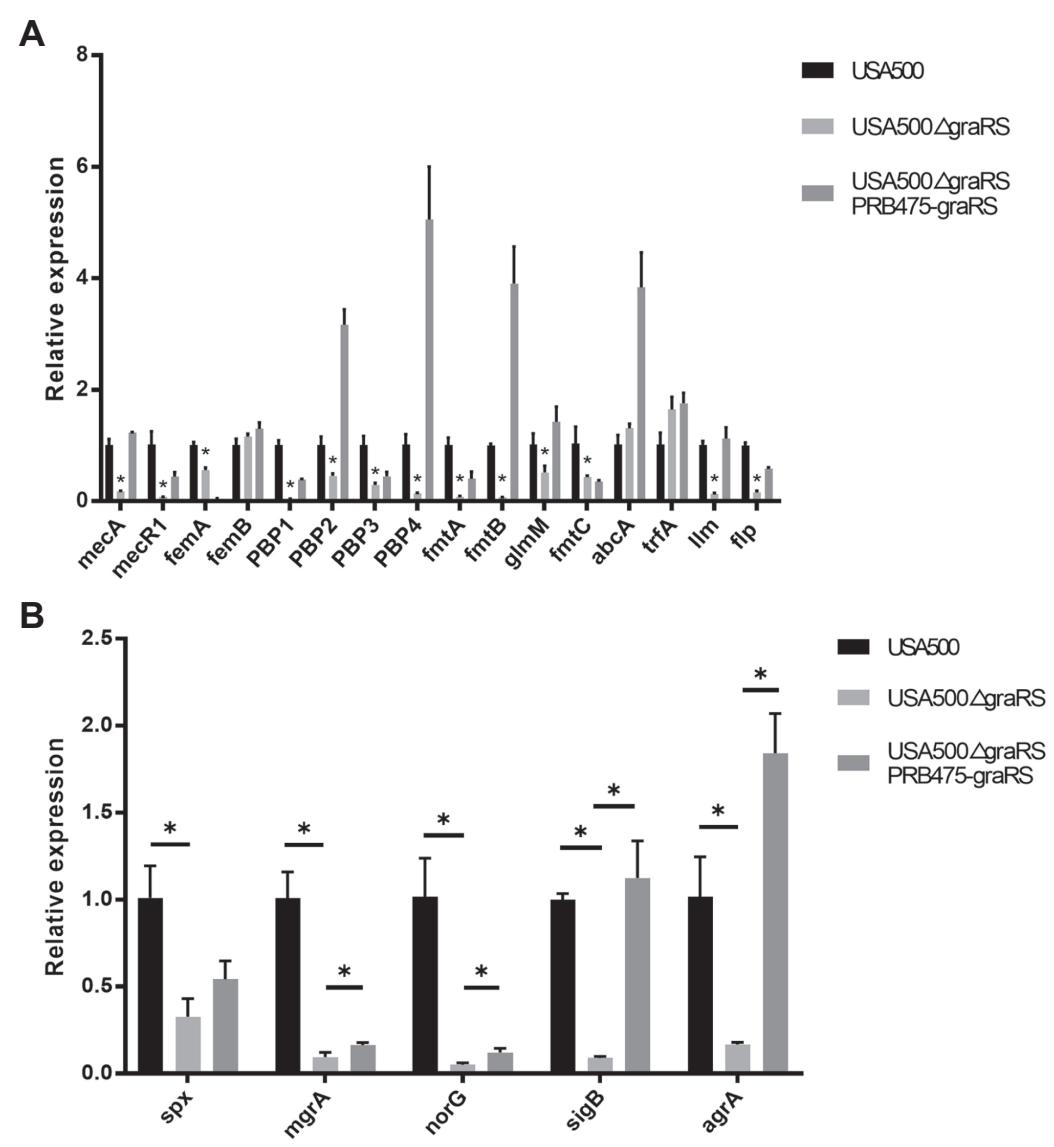
The effect of *graRS* knockout on the mRNA levels of the antibiotic resistance-related genes. The mRNA of the USA500WT, USA500Δ*graRS*, and USA500Δ*graRS::*pRB475-*graRS* strains cultured in TSB at 4 h was extracted, then qRT-PCR was performed to detected the mRNA levels of β-lactam resistance-related genes **(A)** and the antibiotic resistance-related global regulatory genes **(B)**.

According to the bioinformatics analysis, the DEGs in GSE26016 were related to monobactam biosynthesis. Monobactams are beta-lactam antibiotics that contain a monocyclic beta-lactam nucleus and could cause cell wall impairment in MRSA (Liu et al., 2018; Wang et al., 2019). To investigate the effect of *graRS* two-component system on antibiotics resistance, antimicrobial susceptibility test using the broth microdilution method was carried out. The minimum inhibitory concentration (MICs) of ampicillin, oxacillin, vancomycin, and gentamicin for the USA500∆*graRS* mutant were 2, 2, 0.25, and 0.5 μg ml, respectively, while were >8, >8, 1, and 4 μg/ml for the parent strain USA500. The gene complementation by plasmid expressing *graRS* in USA500∆*graRS* restored the MIC values to the wild-type levels (Table 4). In addition, the mRNA levels of β-lactam resistance-related genes were detected by qRT-PCR. The results indicated that the transcriptional levels of the genes encoding penicillin-binding proteins PBP1, PBP2, and PBP4, the PBP2a (or PBP2’) encoding gene *mecA*, peptidoglycan synthesis genes *femA*, autolytic activity related gene *llm*, global regulator genes *mgrA*, *spx*, *agrA*, and *sigB*, the cationic antimicrobial peptide (CAMP) resistance-related genes *dltX*, *dltA*, *mprF*, *vraF*, and *vraG*, were significantly down-regulated in USA500Δ*graRS* mutant strain (Figures 3A,B; Supplementary Figure S2).

**Table 4:** The MIC of ampicillin, gentamicin, oxacillin, and vancomycin for different *Staphylococcus aureus* strains (the broth microdilution method).

	MIC^*^			
	Ampicillin	Oxacillin	Vancomycin	Gentamicin
USA500WT	>8	>8	1	4
USA500Δ*graRS*	2	2	0.25	0.5
USA500Δ*graRS*:: pRB475-*graRS*	8	>8	1	4
ATCC29213	2	0.125	0.5	1

* *MIC: minimum inhibitory concentration*.

## Discussion

The two-component system *graRS* of *S. aureus* belongs to the IM-HK (intramembrane-sensing histidine kinase) family and is conserved within the firmicutes. Previous studies of the *graRS* TCS mainly focused on its regulatory role in the resistance to host defense CAMPs and vancomycin (Kawada-Matsuo et al., 2011; Kolar et al., 2011; Cheung et al., 2014). In recent decades, with the development of high-throughput microarray technology, transcriptome and bioinformatics analysis have been widely used in disease biology areas. Falord, M et al. reported the differentially expressed genes between the *S. aureus* parental HG001 strain and the Δ*graRS* mutant (mid-exponential phase in TSB with 50 μg/ml colistin; Falord et al., 2011), while the detailed description of the interaction between DEGs needs further exploration. In our present study, to make a better understanding of the function of *graRS* in *S. aureus*, the dataset GSE26016, which including three *Staphylococcus aureus* HG001 WT strains and three Δ*graRS* mutant strains, was analyzed in detail. As a result, 563 DEGs were identified between WT group and Δ*graRS* mutant group, including 269 upregulated DEGs and 294 downregulated DEGs (adj. *p* < 0.05, |log_2_FC| > 1). According to GO and KEGG analyses, the DEGs were mostly enriched in pathogenesis and microbial metabolism in diverse environments, respectively (Figures 1C,D). The PPI results showed that GraR and GraS proteins were relevant to MprF and VraS (Figures 1E,H). Meanwhile, WGCNA suggested that the turquoise module was related to the blue module, and the genes in these two modules were associated with *S. aureus* virulence and infection (Supplementary Figures S1A–C; Table 3). The above analyses in GSE26016 showed that the expression levels of some virulence-associated genes were significantly downregulated in Δ*graRS* mutant strains in the condition of 50 μg/ml colistin. In addition, DEGs function enrichment analysis results suggest that *graRS* plays a role in pathogenesis and antibiotic resistance. To make a relatively comprehensive insight into the effect of *graRS* on bacterial virulence phenotypes and antibiotic resistance, in the present study, *S. aureus* USA500 2395 strain was selected and cultured without antibiotic interference. USA500Δ*graRS* showed a weaker β-hemolysis on blood agar plates, and the same trend was shown in its ability of coagulase and pigment production (Figures 2B–D). β-hemolysis is related with Staphylococcal α-hemolysin, a pore-forming toxin, which is encoded by *hla* and expressed in an *agr*-dependent manner. According to the results of qRT-PCR, the transcription of *hla* and *agrA* was significantly downregulated in Δ*graRS* mutant, which is consistent with the weaker β-hemolysis phenotype. Coagulase (encoded by *coa*), another important virulence factor produced by *S. aureus*, is a polypeptide that binds to and activates prothrombin, thereby converting fibrinogen to fibrin and promoting clotting of plasma or blood. We found that *coa* transcription was significantly downregulated in Δ*graRS* mutant, which is correlated with its coagulase production. It has been reported that another toxin, β-toxin, encoded by *hlb* gene, is required for the lethal effect of *S. aureus* culture on silkworm larvae. Our work showed that the transcription level of *hlb* was significantly lower in the Δ*graRS* mutant, compared to that in the USA500 WT strain. We further established the A549 epithelial cell invasion model and the *G. mellonella* infection model to assess the effects of *graRS* on bacterial cell invasion and *in vivo* infection, and the results showed the *graRS* knockout was associated with a decreased number of intracellular bacteria and a higher survival rate of *G. mellonella* (Figures 2H,I). Our data indicate that GraRS plays a vital role in regulating various aspects of *S. aureus* virulence.

Boles et al. (2010) reported that transposon insertion mutations in the *graS* gene of the *S. aureus* strains SH1000 and LAC resulted a significant decrease in biofilm formation in a flow cell apparatus. Consistently, we found that the *graRS* knockout mutation led to reduced biofilm formation, as the microtiter plate assay showed a lower OD_570_ value in the USA500Δ*graRS* than that of the USA500 WT strain and the *graRS* complementation strain (Figures 2E,F). The low expression of *dlt* operons, *ica*, and *atlA* was related to decreased biofilm formation (Herbert et al., 2007; Boles and Horswill, 2008), while *agr* mutation enhanced biofilm formation (Yarwood et al., 2004; Boles and Horswill, 2008; Jenul and Horswill, 2019). In our study, qRT-PCR results showed that the expression of *dltX*, *dltA*, and *agrA* was significantly decreased in USA500Δ*graRS* mutation strain (Supplementary Figure S2; Figure 3B). It could make a combined influence on biofilm formation which needs further exploration.

According to the results of the antimicrobial susceptibility test in this study, the TCS *graRS* knockout in MRSA strain USA500 provides increased sensitivity to ampicillin, gentamicin, oxacillin, and vancomycin, which suggest an important role of *graRS* in regulating antibiotic resistance in MRSA (Table 4). The mRNA levels of the relevant genes including *mprF*, *mgrA*, *spx*, and *vraF* were significantly downregulated in USA500Δ*graRS* mutant strain. Previous studies have shown that *vraFG* (encoding a peptide efflux pump), *mprF* (encoding the MprF enzyme and modulating the charge of cell membrane outer surface), and the *dlt* operon (encoding components modulating the cell wall charge) are regulated by the *graRS* sensory system (Li et al., 2007; Pietiainen et al., 2009; Bayer et al., 2016). The *graRS* system plays an important role in cell wall stress responses (Meehl et al., 2007; Yang et al., 2012). Furthermore, the *graRS* system seems to be important to neutrophil killing, as the *dltX* and *mprF* play an important role in response to cationic antimicrobial peptides (CAMPs; Kraus et al., 2008; Ernst et al., 2009; Rajagopal et al., 2016; Kamar et al., 2017). According to the modulation of anionic charge of cell surface components, *S. aureus* can obtain protection from the harmful effects of the CAMPs activity (Peschel and Sahl, 2006). Meanwhile, *mprF* is a hub gene that confers resistance to daptomycin (Mehta et al., 2012). In some reports, as a global transcriptional regulator, *mgrA* can affect the expression of virulence genes and has a negative regulation in autolysis genes (Trotonda et al., 2009; Sun et al., 2011; Crosby et al., 2016). Bai et al. (2019) have reported that silence *spx* results in a significant increase in oxacillin susceptibility of USA300 and USA500 strains.

In conclusion, the *graRS* knockout in the MRSA USA500 strain repressed bacterial virulence *in vitro*, in the cell model and in the *G. mellonella* infection model. Meanwhile, the Δ*graRS* mutant was more susceptible to various antibiotics. The transcriptional profile analysis data of the Δ*graRS* mutant was consistent with the altered phenotypes. These findings provide novel insights into the role of the *graRS* system in regulating *S. aureus* virulence and antibiotic resistance.

## Data Availability Statement

The original contributions presented in the study are included in the article/Supplementary Material, and further inquiries can be directed to the corresponding author/s.

## Author Contributions

YW, KZ, DQ, and CZ designed the work and revised the manuscript. LC performed bioinformatic analysis. LC, ZW, TX, HG, FZ, XZ, and XL completed all the experiments. LC, ZW, KZ, and YW performed the statistical analysis, made the figures, and wrote the manuscript. All authors contributed to the article and approved the submitted version.

## Conflict of Interest

The authors declare that the research was conducted in the absence of any commercial or financial relationships that could be construed as a potential conflict of interest.

## Publisher’s Note

All claims expressed in this article are solely those of the authors and do not necessarily represent those of their affiliated organizations, or those of the publisher, the editors and the reviewers. Any product that may be evaluated in this article, or claim that may be made by its manufacturer, is not guaranteed or endorsed by the publisher.

## Footnotes

^1^ http://www.ncbi.nlm.nih.gov/geo

^2^ http://string-db.org/cgi/input.pl

## Supplementary Material

SUPPLEMENTARY FIGURE S1 WGCNA analysis and the mRNA levels of interested genes in GSE26016. **(A)** Clustering dendrogram of genes in GSE26016. **(B)** Clustering tree based on the module eigengenes of modules. **(C)** Network heatmap plot in the co-expression modules. **(D)** The mRNA levels of *hla*, *hlb*, and *coa* in GSE26016. (E) The mRNA levels of *mprF*, *dltX*, *dltA*, and *agrA* in GSE26016. WGCNA: weighted gene co-expression network analysis.

SUPPLEMENTARY FIGURE S2 The effect of *graRS* knockout on the mRNA levels of the CAMP resistance-associated genes. The mRNA of the USA500WT and USA500Δ*graRS* cultured in TSB at 4 h was extracted, then qRT-PCR was performed to detected the mRNA levels of the *graR*, *graS*, *vraF*, *vraG*, *dltX*, and *dltA*.
