# Supplementary figures and images for: The Role of graRS in Regulating Virulence and Antimicrobial Resistance in Methicillin-Resistant Staphylococcus aureus
Source: Front Microbiol. 2021 Aug 16;12:727104. doi: 10.3389/fmicb.2021.727104 (PMC8415711; doi:10.3389/fmicb.2021.727104)

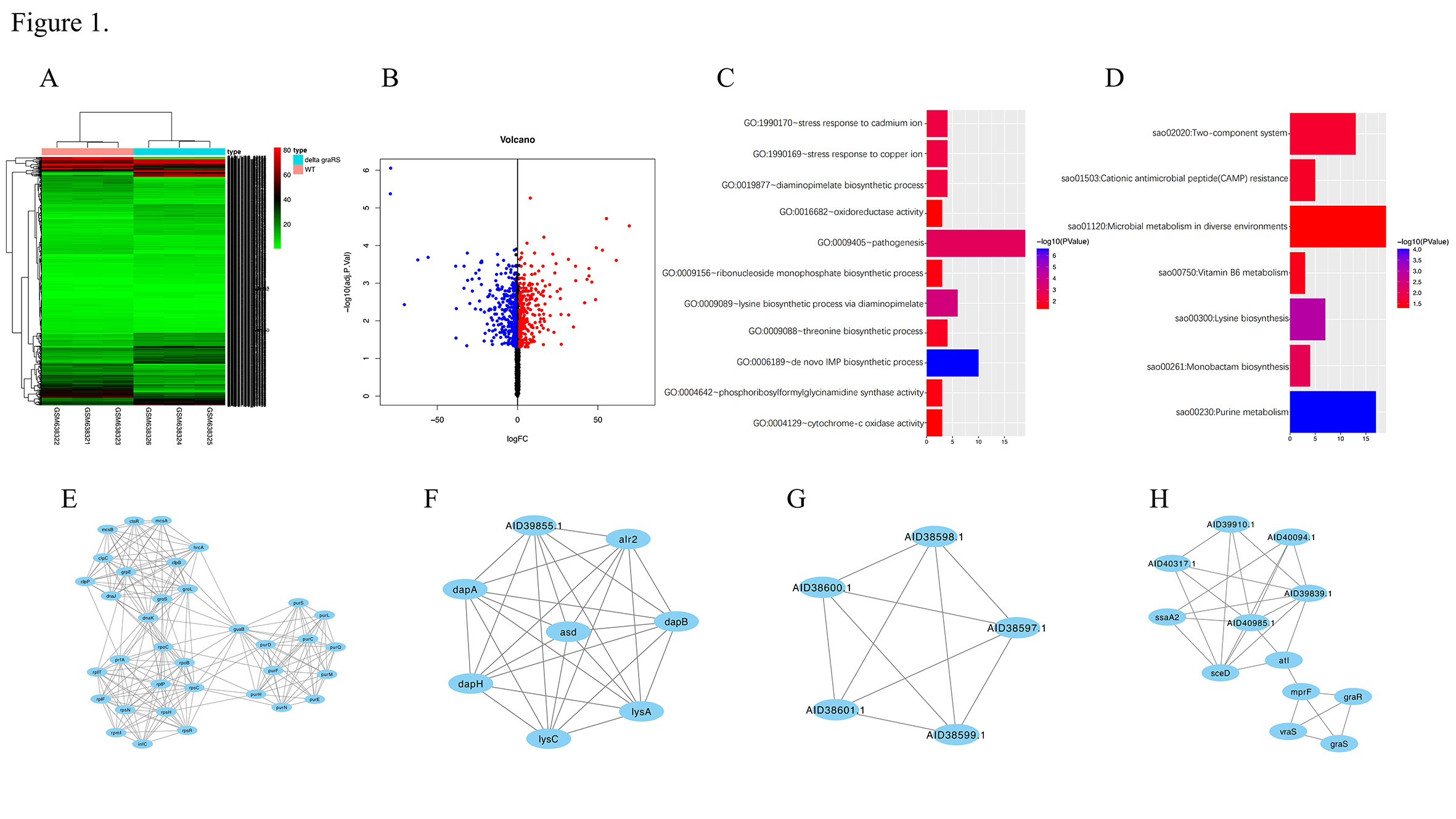

Supplement: SUPPLEMENTARY FIGURE S1 — WGCNA analysis and the mRNA levels of interested genes in GSE26016. (A) Clustering dendrogram of genes in GSE26016. (B) Clustering tree based on the module eigengenes of modules. (C) Network heatmap plot in the co-expression modules. (D) The mRNA levels of hla, hlb, and coa in GSE26016. (E) The mRNA levels of mprF, dltX, dltA, and agrA in GSE26016. WGCNA: weighted gene co-expression network analysis. [file Image_1.JPEG]

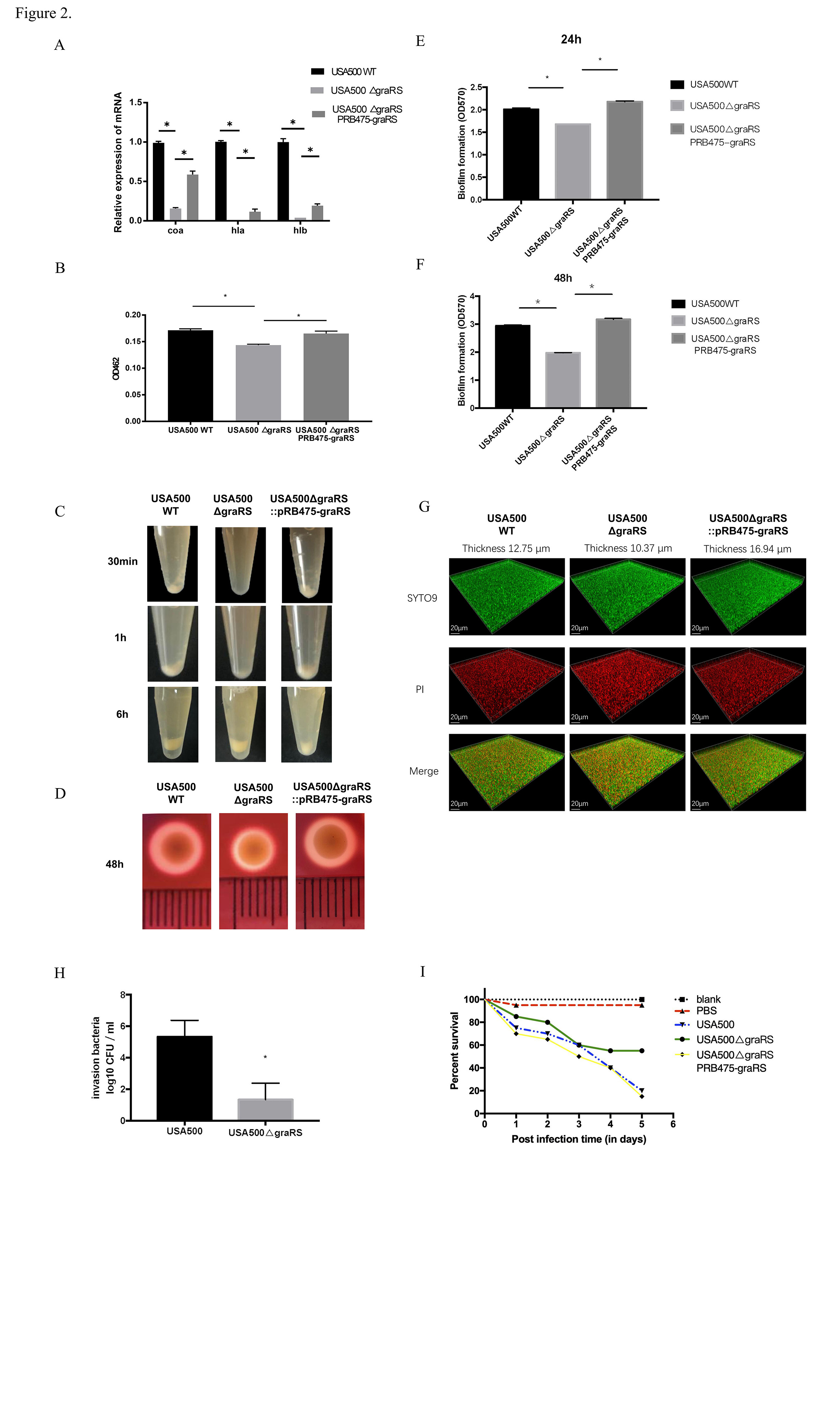

Supplement: SUPPLEMENTARY FIGURE S2 — The effect of graRS knockout on the mRNA levels of the CAMP resistance-associated genes. The mRNA of the USA500WT and USA500ΔgraRS cultured in TSB at 4 h was extracted, then qRT-PCR was performed to detected the mRNA levels of the graR, graS, vraF, vraG, dltX, and dltA. [file Image_2.JPEG]
